# Quality Improvement Project: Introducing Pharmacy Input Into Consultant Psychiatry Outpatient Clinics

**DOI:** 10.1192/bjo.2025.10369

**Published:** 2025-06-20

**Authors:** Mohan Gondhalekar, Ishraq Chowdhury

**Affiliations:** North East London NHS Foundation Trust, London, United Kingdom

## Abstract

**Aims:** This Quality Improvement (QI) Project aimed to enhance the overall level of care received/experienced by patients within the Havering Older Adult Mental Health Team (HOAMHT) through combining the clinical expertise of a Consultant Psychiatrist with the pharmacological acumen of a Specialist Mental Health Pharmacist, within a joint mental health outpatient clinic. Key areas tackled included: medication adherence, faster optimization of psychotropic medications, management of polypharmacy, de-prescription of drugs of dependence, physical health monitoring, and expediting patient discharge from HOAMHT back to the GP.

**Methods:** Our QI project utilised Plan/Do/Study/Act (PDSA) cycles. The first PDSA cycle took place in 2023/2024 over 6 months. The second PDSA cycle took place in 2024/2025 over 6 months. The 1st PDSA Cycle used patient satisfaction outcome scoring, which was randomly collected from 15 patients that had been reviewed within the joint clinics. The results from the 1st PDSA cycle led to a second PDSA Cycle being undertaken, in which the establishment of a ten minute pharmacist’s corner feature was implemented within the joint clinic, and further patient satisfaction data was collected. Based on this data, in 2025/26 a third PDSA cycle will take place over 6 months, where there will be joint clinics consisting of junior doctors and pharmacists. This will serve to develop and refine teaching opportunities for the specialist clinical pharmacists. Then, the 4th PDSA cycle will look to expand and include other community mental health teams within our Trust, in order to see if improvements are possible to be achieved at scale.

**Results:** PDSA Cycle 1: There was a 38% improvement in patient satisfaction scoring for joint clinics vs stand-alone consultant/junior doctor clinics.

PDSA Cycle 2: Patient satisfaction scores increased further with the introduction of stratification, where the pharmacist was given protected time within the clinic to tackle medication-related queries, which patients found invaluable.

**Conclusion:** In England, there is just one Consultant Psychiatrist for every 12,600 people. Hence, the demands on clinical services for treatment have become unsustainable. Consequently, a novel and agile approach is required when organising community mental health services, so that all available clinical knowledge and expertise is exploited and geared towards maintaining a high quality of clinical care for patients, despite the resource limitations that are present. This QI project serves to demonstrate the value of effective collaboration between professionals in the pursuit of clinical excellence.

